# Plasma Brain-Derived Neurotrophic Factor as a Biomarker for the Main Types of Mild Neurocognitive Disorders and Treatment Efficacy: A Preliminary Study

**DOI:** 10.1155/2016/4095723

**Published:** 2016-08-11

**Authors:** Oleg A. Levada, Nataliya V. Cherednichenko, Andriy V. Trailin, Alexandra S. Troyan

**Affiliations:** State Institution “Zaporizhzhia Medical Academy of Postgraduate Education Ministry of Health of Ukraine”, 20 Winter Boulevard, Zaporizhia 69096, Ukraine

## Abstract

Decreased levels of brain-derived neurotrophic factor (BDNF) are assumed to play a crucial role in the pathophysiology of mild neurocognitive disorders (MNCDs). In this study, we compared plasma BDNF levels (at baseline and after two months of treatment with escitalopram) in patients with the main types of MNCDs and normal controls. 21 patients met the DSM-5 diagnostic criteria for possible MNCD due to Alzheimer's disease (MNCD-AD); 22 patients fulfilled the diagnostic criteria for subcortical vascular MNCD (ScVMNCD) according to Frisoni et al. (2002) and neuroimaging-supported probable diagnosis of vascular MNCD according to DSM-5; 16 subjects entered control group. At baseline, we detected lower BDNF levels in both MNCD groups, which was significant only in subjects with MNCD-AD. Moreover, plasma BDNF level of 21160 pg/mL showed high sensitivity (94%) to discriminate patients with MNCD-AD. Decreased plasma BDNF highly correlated with the severity of memory impairment and total MMSE score in MNCD-AD group. Escitalopram treatment in patients with MNCD-AD or ScVMNCD led to an increase of plasma BDNF concentrations and as a result to a decrease of cognitive, depressive, and anxiety symptom severity. In conclusion, plasma BDNF might be a reliable biomarker for the validation of MNCD-AD diagnosis and treatment efficacy.

## 1. Introduction

Mild neurocognitive disorders (MNCDs) as an intermediate stage between normal cognitive aging and dementias, particularly Alzheimer's disease (AD), have recently become a subject of an increasing scientific interest [1]. This interest arises from the perspective of significant medical and social value and potential capability to prevent MNCD conversion into different types of dementias (major neurocognitive disorders). The diagnostic construct of MNCD is substantially congruent with the previously proposed nosological entity for mild cognitive impairment (MCI) [2]. It was shown that overlap between MNCD and MCI diagnosis is 98.6% [3].

According to recent epidemiological data, the overall prevalence of MNCDs among individuals older than 55 is 15.7%, with single-domain amnestic, multiple-domain amnestic, and nonamnestic subtype prevalence of 6.4%, 3.7%, and 5.6%, respectively [4]. Amnestic variants share about 65% in the structure of MNCDs [5]. The main etiological type of amnestic MNCDs is MNCD due to Alzheimer's disease (MNCD-AD) [6]. The second common etiological type of MNCD is subcortical vascular one (ScVMNCD) with the prevalence of 37.3% [7]. ScVMNCD manifests with clinical symptoms of subcortical vascular dementia, though the severity of the impairment does not reach the level of dementia and cognitive deficit does not interfere with the capacity for independence in everyday activities [8].

The investigation of neurobiological aspects of MNCDs might shed light on some pathogenetic mechanisms, which could become targets for management of MNCDs. The expression of growth factors, in particular brain-derived neurotrophic factor (BDNF), is one of them. In the majority of neurodegenerative and vascular dementias a reduction of BDNF concentration in the brain and concurrently in plasma [9] or serum has been reported [10, 11]. Moreover, an increase of BDNF expression has been observed in patients taking selective serotonin reuptake inhibitors (SSRIs) and antidement drugs [10]. Therefore, we could assume that the decrease of plasma/serum BDNF level might be used as a biological marker of MNCD's diagnosis, whereas the increase of this neurotrophin might be used for the assessment of treatment efficiency.

Limited information is available for plasma/serum BDNF concentrations in patients with MNCDs. Although low levels of BDNF in serum [12] and plasma [13] were found in patients with MNCD-AD, no studies are available concerning BDNF levels in patients with ScVMNCD so far. Therefore, a comparative study of plasma BDNF levels in different etiological types of MNCDs seems to be relevant. The current lack of effective MNCD's treatment based on a high-level evidence warrants a search for new approaches based on neuroprotective strategies.

Hence, the purpose of our study was to evaluate plasma BDNF concentrations in patients with the main etiological types of MNCDs and to determine whether the assessment of plasma BDNF level could improve the diagnostics of MNCD-AD and ScVMNCD. We also aimed to study the dynamics of plasma BDNF in MNCD-AD/ScVMNCD patients after escitalopram treatment. We chose escitalopram taking into account the evidence about its stimulation of BDNF expression [14], a frequent comorbidity of MNCDs and depressive or/and anxiety disorders [15], as well as the priorities of escitalopram efficacy and safety in this clinical setting [16].

## 2. Materials and Methods

### 2.1. Subjects and Procedures

59 persons over 65 years were enrolled in the study. 21 patients met the diagnostic criteria for possible MNCD-AD according to DSM-5 [2]. 22 patients fulfilled the diagnostic criteria for ScVMNCD according to Frisoni et al. [8] and probable neuroimaging-supported diagnosis of vascular MNCD according to DSM-5 [2]. 16 subjects were recruited in a control group without cognitive impairment (WCI). This study was approved by the local ethics committee and was performed in accordance with the ethical standards laid down in the 1964 Declaration of Helsinki and its later amendments. All participants gave written informed consent prior to participation in the study.

### 2.2. Assessments

Clinical protocol included the following: (1) collection of anamnestic data; (2) neuropsychological testing: MMSE [17], Luria's tests [18], study of memory (TIME test) [19], clock drawing test [20], and verbal fluency test [21]; (3) neuropsychiatric assessment using the Neuropsychiatric Inventory (NPI) [22]; (4) neurological examination with detailed assessment of walking, Tinetti Performance Oriented Mobility Assessment (POMA) [23]; and (5) the assessment of daily living activities, BADL [24]. The severity of impairments was evaluated on a scale with a range from 0 to 3 (where 0 meant “not impaired” and 3 meant “the most impaired”) or according to authors' recommendations to the scales. Patients underwent MRI or CT brain scan to fulfill the criteria of MNCD-AD and ScVMNCD.

### 2.3. Biochemical Investigation

BDNF levels were measured by the ELISA method (kit supplied by R&D Systems, Inc., Minneapolis, USA), according to the manufacturer's protocol using the immunoassay photometer “ImmunoChem-2100” (USA). Clinical studies and the evaluation of plasma BDNF concentrations were performed twice: at baseline in all enrolled persons and after 2 months of escitalopram treatment (daily dosage: 10 mg) in 20 patients with MNCDs. This dosage was prescribed in accordance with the FDA instruction for using the drug in elderly patients.

### 2.4. Statistical Analysis

Statistical analyses were conducted using “STATISTICA 6.0” for Windows (StatSoft Inc., USA) v.6.1 and SPSS (version 19.0, SPSS Inc., Chicago, USA). The results were given as percentage, median, and interquartile range or mean and standard deviation, depending on the data distribution. The statistical significance of between-group comparisons was determined using parametric and nonparametric criteria when appropriate (Kruskal-Wallis test with subsequent multiple comparisons, Mann-Whitney test, Wilcoxon test, *χ* ^2^ test, ANOVA, post hoc Scheffe test, and *t*-test). The relationships between clinical variables and plasma BDNF levels were assessed using Spearman's (*r*_*s*_) or Pearson's (*r*) correlation coefficients. In addition, we calculated the areas under the receiver operating characteristic curves (AUC-ROC) to determine the value of plasma BDNF concentrations to discriminate patients with MNCD-AD and SсVMNCD. A cutoff was derived from ROC curve to yield empirical optimal sensitivity and specificity. Significance for the results was set at *p* < 0.05.

## 3. Results 

The main demographic, cognitive, neuropsychiatric, neurological, and functional features of the comparison groups are summarized in Table 1. Surveyed cohorts did not differ by age, gender, and level of education. The severity of cognitive impairments (total MMSE score) corresponded to the recommended rates for those with MNCD. It was significantly higher in both MNCD-AD and SсVMNCD groups in comparison with control. Meanwhile, we found no significant difference in cognitive impairment between MNCD-AD and SсVMNCD groups.

The analysis revealed some significant clinical features that reliably distinguished patients with MNCD-AD and SсVMNCD. The most prominent feature of MNCD-AD patients was amnestic syndrome, which was significantly more severe in comparison with WCI. The severity of impairment of spontaneous delayed recall of five nouns was comparable in MNCD-AD and ScVMNCD groups. However, patients with MNCD-AD did not significantly improve the results of recall after cues. Subjects with MNCD-AD also had mild visuospatial apraxia when performing Luria's tests. This symptom differentiated MNCD-AD patients from controls and those with ScVMNCD, who did not have any visuospatial disturbances. We also found no significant neurological disturbances in MNCD-AD group. Similarly, patients with ScVMNCD showed distinct spontaneous delayed recall impairment, but they had significantly higher rates of cued verbal recognition than those with MNCD-AD. In ScVMNCD group, we revealed pronounced executive and neurological disturbances in contrast to the WCI and MNCD-AD subjects. The executive dysfunction manifested in performing clock drawing test (part I), verbal fluency test, and Luria's tests for kinetic apraxia. Moreover, there were mild to moderate pseudobulbar signs and frontal lobe gait disturbances (decreased POMA score) in ScVMNCD patients. The total score of psychopathological impairments (NPI) of ScVMNCD group was significantly higher than for WCI group, whereas the NPI score between MNCD-AD and WCI groups differed only at the level of trend. Predominant psychopathological symptoms in WCI group were anxiety (93.8%), insomnia (75.0%), and irritability/emotional instability (56.3%). In MNCD-AD group we observed anxiety and sleep disturbances (by 90.5%), irritability/emotional instability (66.7%), and depression (61.9%), whereas in ScVMNCD group we observed apathy (90.9%), depression, anxiety, and sleep disturbances (by 86.4%).

On average, MNCD patients had minimal disruption in everyday activities by BADL scale; however, it was significantly different from controls.

Table 2 provides plasma BDNF concentrations in three comparison groups. At baseline, we detected reduced plasma BDNF levels in both MNCD groups. Nevertheless, only in MNCD-AD group the reduction was significant compared with control.

Next, we determined possible correlations between plasma BDNF concentrations and distinguishing clinical features of MNCD-AD and ScVMNCD groups described above (Table 3).

We found highly significant positive correlations between plasma BDNF levels and the score of spontaneous delayed recall (TIME test, five unrelated nouns) in MNCD-AD group, as well as moderate positive correlations between plasma BDNF levels and general cognitive functioning (MMSE total score). Plasma BDNF concentrations negatively associated with visuospatial apraxia and impairment in daily living activities (BADL score).

As for the ScVMNCD group, plasma BDNF concentrations significantly correlated only with spontaneous word recall and visuospatial deficit. These relationships were less significant in comparison with the MNCD-AD group. We observed no significant correlations between plasma BDNF and psychopathological impairment or patient's age in both MNCD groups. Hence, the reduction of BDNF levels predominantly influenced the severity of the amnestic syndrome in MNCD-AD patients and consequently the overall severity of cognitive and daily living impairment.

**Figure 1:**
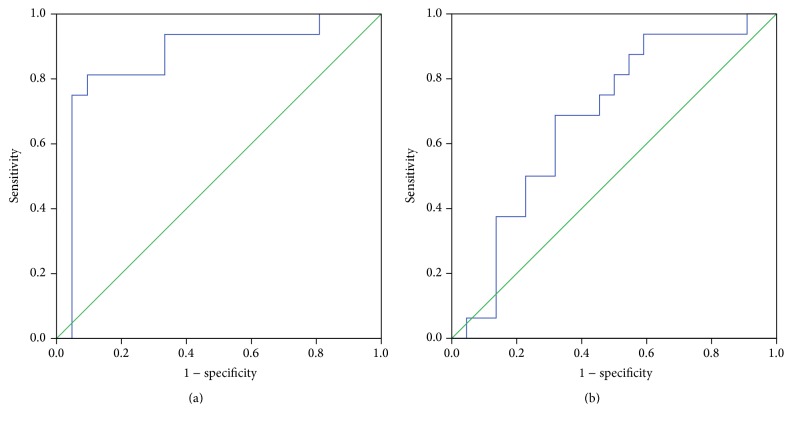
ROC curves for plasma BDNF to diagnose MNCD-AD (a) and ScVMNCD (b). AUCs (95% confidence intervals) are 0.866 (0.733–0.999) for MNCD-AD (*p* < 0.001) and 0.679 (0.506–0.852) for ScVMNCD (*p* = 0.063). ROC receiver-operating characteristic. AUC area under the curve. MNCD-AD mild neurocognitive disorder due to Alzheimer's disease. ScVMNCD subcortical mild neurocognitive disorder.

The discriminating ability of plasma BDNF for the MNCD-AD/ScVMNCD and control was determined by the AUC analysis.  Figure 1 shows the receiver operating characteristic (ROC) curve for plasma BDNF in the diagnoses of MNCD-AD and ScVMNCD. The analysis of ROC curve yielded AUC of 0.866 (95% CI: 0.733–0.999) for MNCD-AD indicating good diagnostic value (*p* < 0.001). We derived arbitrarily the cut-off value of 21160 pg/mL from the coordinates of ROC curve as having optimal performances in discrimination between MNCD-AD and WCI persons. This plasma BDNF level showed high sensitivity (94%) and moderate specificity (67%). On contrary, plasma BDNF level exhibited poor discriminatory power in predicting the diagnosis of ScVMNCD: AUC 0.679 (95% CI: 0.506–0.852); *p* = 0.063 (Figure 1).

After two months of escitalopram treatment, 20 patients (10 in each MNCD group) underwent repeated clinical examination and evaluation of plasma BDNF concentrations. The results are shown in Table 4.

The intake of escitalopram increased plasma BDNF concentrations in both MNCD groups; nevertheless, those differences did not reach statistical significance. Simultaneously, escitalopram treatment improved clinical parameters in patients with MNCD-AD and ScVMNCD. Thus, we observed a significant increase in general level of cognitive functioning (MMSE total score) and a decrease in the frequency and severity of neuropsychiatric symptoms (mainly depression and anxiety) by NPI scale. We detected significant improvement in cognitive functioning in the MNCD-AD group, predominantly in memory domain (spontaneous delayed recall). At the same time, patients with ScVMNCD mainly improved executive functions (verbal fluency test, clock drawing test, and kinetic praxis), although the results were not statistically significant. The overall score of impairment in daily living activities (BADL score) decreased at the level of trend in both MNCD groups.

## 4. Discussion

In this study, we found a decrease in plasma BDNF concentrations in patients with the main etiological types of MNCDs. The decrease was significant in patients with MNCD-AD and correlated with the severity of amnestic syndrome. Plasma BDNF was found to be highly sensitive in distinguishing MNCD-AD patients from WCI subjects. After escitalopram treatment, we observed an increase in plasma BDNF levels in both MNCD groups and simultaneous improvement of distinguishing clinical features of MNCD-AD and ScVMNCD. Moreover, we found a good accuracy of plasma BDNF in discriminating patients with MNCD-AD and persons WCI. Our results are consistent with findings for amnestic MNCD obtained by Hwang and colleagues [13]. To the best of our knowledge, this is the first study of plasma BDNF levels in patients with ScVMNCD. Decreased plasma/serum BDNF levels have been previously reported only in different morphological types of vascular dementia [9, 10].

As it was shown, brain neurodegenerative processes at the early AD stages could affect neurons and glial cells in the main areas of BDNF synthesis, such as the hippocampus, amygdala, and neocortex [25]. Therefore, our data suggest that low plasma BDNF concentrations in patients with MNCD-AD can be the direct sequence of decreased brain BDNF expression. Unlike neurodegeneration, microvascular process in ScVMNCD might not have such a marked influence on the sites of BDNF expression. However, the severity of nervous tissue damage in manifest vascular dementia might sufficiently decrease the BDNF synthesis [10].

The link between decreased BDNF concentrations and the severity of amnestic syndrome indicates a key role of BDNF in memory impairment among MNCD-AD patients. Jovanovic et al. reported that BDNF increased the opening of NMDA receptors and enhanced expression of AMPA receptors of the postsynaptic membrane, resulting in facilitating of long-term potentiation (LTP) [26]. LTP is a form of synaptic plasticity that represents a cellular model for learning and memory. It strengthens the synaptic transmission between two neurons, which remains for a long time after exposure to synaptic pathway. BDNF plays an important role in the late phase of LTP, which lasts at least eight hours after stimulation [27]. Furthermore, BDNF is involved in axons' branching and dendrites' growth of glutamatergic neurons [28]. That increases the density of glutamatergic synapses in hippocampal and neocortical structures. Thus, not only is reduced serum BDNF level an early marker of cognitive impairment of neurodegenerative etiology, but it also reflects pathogenetic aspects of neurocognitive disorders, such as the reduced support of synaptic plasticity underlying in memory process.

The ability of plasma BDNF level to discriminate patients with MNCD-AD makes it a candidate biomarker for early identification, monitoring, and intervention assessment in such an etiological type of MNCDs.

Our study demonstrated that escitalopram treatment (10 mg per day) led to an increase in plasma BDNF levels in both MNCD-AD and ScVMNCD groups. The increase of plasma BDNF levels correlated with a significant improvement in cognitive functioning and reduction of neuropsychiatric symptoms (predominantly depression and anxiety) in both MNCD groups as well as with significant amelioration in memory performance in MNCD-AD patients and uncertain improvement in executive functions in ScVMNCD persons. According to Ladea and Bran, treatment with escitalopram can significantly reduce the severity of depressive symptoms and simultaneously increase serum BDNF concentrations in elderly patients [29]. Meanwhile, our study demonstrated a positive effect of escitalopram on cognitive functioning in patients with MNCD, mainly in the memory domain. Probably, the impact of escitalopram is mediated by the raise of the expression of BDNF in hippocampal and neocortical brain structures, which in turn leads to normalization of glutamate-dependent mechanism of synaptic plasticity.

This study has some limitations. As a preliminary study, our sample size was relatively small. Another limitation is that the ELISA kit we used quantified the total BDNF concentrations without distinction between pro-BDNF and mature BDNF variants. Furthermore, our findings are based on the concept that plasma BDNF level is associated with alterations in the brain [30]. Although peripheral BDNF might originate from non-brain tissues, muscle, thymus, heart, liver, lung, spleen, and vascular smooth muscle cells [31, 32], positive correlations have been found between concentrations of BDNF in cerebrospinal fluid and plasma [33]. Since there were few studies that investigated BDNF levels in patients with MNCDs, our results need confirmation in larger samples with control for non-brain sources of BDNF and measurement of two BDNF variants.

## 5. Conclusions

In conclusion, our study demonstrated a decrease of plasma BDNF concentrations in patients with the main MNCDs' etiological types, which was more significant in subjects with MNCD-AD. In patients with MNCD-AD, the reduction of plasma BDNF concentrations was predominantly associated with the memory process impairment. Plasma BDNF levels showed high sensitivity for detecting MNCD-AD. Escitalopram treatment in patients with MNCD-AD and ScVMNCD led to an increase in plasma BDNF concentrations and, as a result, to a decrease of cognitive deficits, as well as to a decrease of depressive and anxiety symptom severity. Plasma BDNF concentrations might be used as a reliable biomarker for the validation of MNCD-AD diagnosis and assessment of treatment efficacy.

## Figures and Tables

**Table 1:** Main demographic, cognitive, psychiatric, neurological, neuropsychiatric, and functional characteristics in comparison groups.

Variables	Comparison groups			*p* value		
	WCI (*n* = 16)	MNCD-AD (*n* = 21)	ScVMNCD (*n* = 22)	WCI versus MNCD-AD	WCI versus ScVMNCD	MNCD-AD versus ScVMNCD
Age (years)	72.06 ± 5.25	73.90 ± 5.52	74.14 ± 5.69		0.478^a^	
Gender, male/female	4/12	8/13	9/13	0.399^b^	0.307^b^	0.850^b^
Education (years)	12.88 ± 2.70	11.67 ± 4.51	12.59 ± 3.83		0.594^a^	
MMSE, score	28 (28-29)	26 (26-27)	26 (24–27)	0.000008	0.000001	0.93
Delayed recall, TIME test, number of words (0–5)	4 (4-5)	2 (1-2)	2 (2-3)	<0.00001	0.0001	0.34
Delayed recognition, TIME test, number of words (0–10)	9 (8–10)	5 (5–7)	8 (7–9)	<0.00001	0.04	0.0004
Clock drawing test, part I, score (0–10)	9 (9-10)	9 (9-10)	7.5 (7-8)	0.46	0.00001	0.0019
Verbal fluency, number of words during 3 min.	19 (15–25.5)	15 (12–21)	13.5 (10–17)	0.51	0.007	0.22
Kinetic apraxia, Luria's tests, score (0–3)	0 (0-0)	0 (0-0)	2 (1–3)	1.0	0.00001	0.00001
Visuospatial apraxia, Luria's tests, score (0–3)	0 (0-0)	1 (0-1)	0 (0-1)	0.019	0.89	0.19
The severity of pseudobulbar syndrome, score (0–3)	0 (0-1)	0 (0-1)	2 (1-2)	1.0	0.00002	0.00001
POMA, score (0–28)	27.5 (26–28)	26 (25-26)	17.5 (13–21)	0.06	<0.00001	0.00002
NPI, score (0–144)	7 (4–10)	11 (8–14)	12.5 (8–17)	0.13	0.025	1.0
Depression, NPI (*n*, %)	5 (31.3)	13 (61.9)	19 (86.4)	0.07^b^	<0.0001^b^	0.07^b^
Anxiety, NPI (*n*, %)	15 (93.8)	19 (90.5)	19 (86.4)	0.71^b^	0.72^b^	0.67^b^
Apathy, NPI (*n*, %)	0 (0)	9 (42.9)	20 (90.9)	0.003^b^	<0.0001^b^	0.001^b^
Irritability, NPI (*n*, %)	9 (56.3)	14 (66.7)	10 (45.5)	0.52^b^	0.51^b^	0.16^b^
Sleep, NPI (*n*, %)	12 (75.0)	19 (90.5)	19 (86.4)	0.21^b^	0.37^b^	0.73^b^
BADL, score (0–60)	0 (0-0)	1 (1-1)	2 (1-2)	0.0001	<0.00001	0.15

Mean ± standard deviation/median (lower/upper quartile) values are presented.

WCI patients without cognitive impairment.

MNCD-AD patients with mild neurocognitive disorder due to Alzheimer's disease.

ScVMNCD patients with mild subcortical vascular neurocognitive disorder.

*n*: number of patients.

^a^One-way ANOVA.

^b^ *χ* ^2^ test.

Nonparametric ANOVA for multiple comparisons if not otherwise specified.

**Table 2:** Plasma BDNF levels (mean ± SD) in comparison groups at baseline, pg/mL.

Comparison groups			*p* value		
WCI (*n* = 16)	MNCD-AD (*n* = 21)	ScVMNCD (*n* = 22)	WCI versus MNCD-AD	WCI versus ScVMNCD	MNCD-AD versus ScVMNCD
31581.5 ± 8092.2	19950.7 ± 9678.8	25939.6 ± 10410.5	0.0025	0.21	0.13

*n*: number of patients.

*p* according to post hoc Scheffe test.

**Table 3:** Spearman's/Pearson's correlations between clinical, demographic, and cognitive variables and plasma BDNF concentrations in MNCD-AD and ScVMNCD groups.

Clinical variables	Spearman's/Pearson's correlation coefficient	
	MNCD-AD group	ScVMNCD group
Age	−0.09	−0.28
MMSE, total score	0.49^*∗*^	0.18
Delayed recall, TIME test	0.72^*∗*^	0.41^*∗*^
Clock drawing test, part I, score	0.21	0.21
Verbal fluency, number of words	0.32	0.09
Kinetic apraxia, Luria's tests, score	−0.16	−0.35^*∗*^
Visuospatial apraxia, Luria's tests, score	−0.34^*∗*^	−0.33^*∗*^
NPI, total score	−0.22	−0.12
NPI, the severity of depression, score	−0.17	−0.22
BADL, score	−0.58^*∗*^	−0.25

^*∗*^ *p* < 0.05.

**Table 4:** The dynamics of plasma BDNF concentrations and main clinical characteristics in MNCD patients taking escitalopram.

Variables	MNCD-AD group (*n* = 10)			ScVMNCD group (*n* = 10)		
	Before treatment	After treatment	*p*	Before treatment	After treatment	*p*
Plasma BDNF concentrations, pg/mL	20660.4 ± 12774.6	26356.0 ± 8309.1	0.15^*∗*^	26652.4 ± 12435.7	30066.0 ± 10796.4	0.27^*∗*^
MMSE, score	26.5 (26-27)	28 (28-29)	0.005	25 (24–26)	27 (26–28)	0.005
Delayed recall, TIME test, number of words (0–5)	2 (1-2)	3.5 (3–5)	0.02	2.5 (2-3)	3 (1–4)	0.83
Verbal fluency, number of words during 3 min.	19.5 (15–25)	17.5 (12–24)	0.62	13 (10–17)	17 (10–19)	0.20
Clock drawing test, part I, score (0–10)	9 (8–10)	9 (8–10)	0.55	7 (7-8)	7.5 (6–9)	0.23
Kinetic apraxia, Luria's tests, score (0–3)	0 (0-1)	0 (0-0)	0.22	2 (1–3)	2 (1-2)	0.48
NPI, total score (0–144)	11.5 (9–14)	6.5 (6–8)	0.0078	16.5 (9–18)	10.5 (7–13)	0.011
POMA, score (0–28)	25 (24–26)	25 (24–26)	1.0	15.5 (13–21)	16 (15–21)	0.043
BADL, score (0–60)	1 (1-1)	1 (0-1)	0.11	2 (1-2)	2 (1-2)	0.98

Data are presented as mean ± standard deviation/median (lower/upper quartile).

*n*: number of patients.

*p*, respectively: ^*∗*^according to *t*-test; by Wilcoxon criterion if not otherwise specified.
